# Fibula allograft in complex three-part and four-part proximal humeral fractures in active patients, a matched case-control study

**DOI:** 10.1016/j.jseint.2023.10.004

**Published:** 2023-11-17

**Authors:** Floortje Lodewika J. Opperman, Leanne S. Blaas, Merel Pape, Nikki Buijs, Maayke v Sterkenburg, Jian Zhang Yuan, Charlotte M. Lameijer, Robert Jan Derksen

**Affiliations:** a Department of Surgery, Amsterdam UMC, location VUmc, Amsterdam, The Netherlands; b Department of Surgery, Zaandam Medical Center, Zaandam, The Netherlands; c Department of Surgery, Rode Kruis Ziekenhuis, Beverwijk, The Netherlands

**Keywords:** Proximal humeral fracture, Medial hinge, Avascular necrosis, Fibula allograft, Locking plate, Functional outcomes, Patient reported outcome measures

## Abstract

**Background:**

About 20% of proximal humerus fractures (PHFs) are unstable and/or markedly displaced and therefore require surgery. Locking plate fixation after anatomical reduction has become the current treatment of choice for these fractures in the active population. However, studies have shown complication rates up to 36%, such as loss of reduction and avascular necrosis. To date, data from literature are inconclusive on outcomes following the use of an intramedullary fibula allograft in PHFs, possibly due to the case mix. It is hypothesized that the use of a fibula allograft is beneficial to prevent secondary displacement of the fracture in cases where the medial hinge is markedly displaced and unstable, resulting in better clinical and patient reported outcomes.

**Methods:**

In this multicenter matched cohort study, patients with an unstable, displaced PHF, including anatomic neck fractures and significantly displaced surgical neck fractures, were included. Patients that were treated with a locking plate augmented with a fibula allograft were matched to patients who had undergone locking plate reconstruction without the allograft. The matches were made based on fracture characteristics, age, and performance status. Functional outcomes, Patient Reported Outcome Measures, complications, and radiographic results were compared.

**Results:**

Twelve patients with fibula allograft augmented osteosyntheses were included and matched to 12 control patients. The mean age was 58 years in the fibula allograft group compared to 62 years in the control group. Minimum follow-up was 12 months. Disability of the Arm Shoulder and Hand score, Constant Shoulder score, abduction, and external rotation were significantly better in the fibula allograft group (17.4 ± 8.6 vs. 26.1 ± 19.2, *P* = .048; 16.5 ± 11.5 vs. 19.8 ± 16.5 *P* = .040; mean 127° ± 38° vs. mean 92° ± 49° *P* = −.045; 50° ± 21° vs. mean 26° ± 23°, *P* = .004). There was no statistically significant difference in the Oxford Shoulder score between groups (*P* = .105). The Visual Analog Scale was not significantly different between groups (3.1 ± 1.8 vs. 1.6 ± 1.9, *P* = .439). Radiographic union was reached in 11 patients of the fibula allograft group compared to 8 in the control group (*P* = .317). The complication rate was twice as high in the control group (3 vs. 7).

**Conclusion:**

Additional support of the medial hinge in unstable PHFs with a locking plate in combination with a fibula allograft appears to create a more stable construct without compromising the viability of the articular surface of the head. The use of a fibula allograft in selected complex cases could therefore result in better clinical outcomes with lower complication rates.

Proximal humeral fractures (PHFs) account for 5% of all fractures. Approximately 80% of these fractures can be treated conservatively if displacement and angulation of fracture parts do not exceed generally accepted margins.^10^^,^^25^ The remaining 20% of PHFs, are considered complex injuries and are considered eligible for surgical treatment.^10^^,^^17^

Anatomical reconstruction and fixation is recommended for patients with complex and displaced multipart fractures in active patients. Proximal humeral locking plate reconstruction has become the gold standard to achieve this goal.^16^ The locking plate construct relies on biomechanical properties providing divergent and convergent fixed-angle screws that improve fixation and pull-out strength in the bone.^10^^,^^25^^,^^38^ Fixed-angle screws are designed to hold the articular part of the humeral head in place, to prevent secondary displacement.^9^^,^^33^^,^^37^

Complication rates as high as 36% after locking plate reconstruction of PHFs have been reported.^3^^,^^8^^,^^10^^,^^12^^,^^16^^,^^27^ The most common complications include secondary loss of reduction due to screw cut-out, implant failure, and/ or subsequent varus collapse of the humeral head, and avascular necrosis (AVN), seen predominantly in complex PHFs.^20^^,^^23^^,^^27^ Displaced anatomical neck PHFs are at risk for developing AVN.^8^^,^^18^ Displacement of the articular segment leads to injury to the circumflex arteries, which may compromise the arterial blood supply and may result in AVN, even after reduction and surgical fixation of the fracture with a locking plate.^7^^,^^18^^,^^26^^,^^32^ Furthermore, most anatomical neck fractures of the proximal humerus are oblique and therefore subject to vertical shear, making reconstructions with mere lateral plate fixation susceptible to secondary displacement and varus collapse.^7^

In unstable complex anatomical neck fractures, the use of a locking plate alone might just not provide enough stability, and failure of the construct is more likely to occur.^4^^,^^9^^,^^33^ Biomechanically this is explained by the short distance from the anatomical neck fracture line to the chondral surface, where only the screw tips are supporting the reduced humeral head. Possible methods to strengthen the anchorage of the implant are to use hollow cement augmented screws to reduce the risk of cut-out and subsequently avoid the loss of reduction and to use nonabsorbable sutures through the rotator cuff and locking plate to distribute the working forces over the construct rather than the fracture site itself.^4^^,^^33^ Above all, careful anatomical reconstruction of the medial hinge is mandatory to prevent varus malalignment of the head and overall provides extra stability to the construct.^24^ In addition, a potential solution to prevent secondary displacement and development of AVN could be aided by adequate medial hinge support using an intramedullary fibula allograft.^25^^,^^26^

Literature to date is inconclusive on the benefits of fibula allograft augmentation in PHFs.^13^ However, in several studies, good clinical and radiographic outcomes and diminished complication rates have been reported when using intramedullary fibula allografts in complex displaced PHFs.^3^^,^^12^^,^^13^^,^^19^^,^^23^^,^^28^^,^^30^^,^^38^ When used as an intramedullary bone peg, the fibula allograft acts like a medial strut perpendicular to the fracture. It supports the articular head, which prevents varus collapse and creates a stable construct by supporting the medial hinge without compromising the viability of the articular surface.^3^^,^^9^^,^^16^^,^^25^^,^^38^ Heterogeneous inclusion criteria regarding age and type of PHF are reported and this results in difficult generalization regarding which patients should be indicated for fibular allograft augmentation of PHFs.

We think that fibula strut augmentation only benefits patients with complex three-part and four-part PHFs. Therefore, we hypothesize that fibula graft augmentation will have better functional outcomes than reconstruction without fibula graft in active patients with complex PHFs.

The purpose of this study is to evaluate the functional and radiological outcomes of a locking plate construct with and without fibular allograft augmentation in active patients with a three-part or four-part PHF and an anatomical neck fracture.

## Patients and methods

In this multicenter, matched cohort study, patients with a complex PHF with involvement of the anatomical neck were included retrospectively. Patients with a locking plate construct and fibula allograft, indicated by surgeons preference, were matched to patients with comparable fracture characteristics, which were treated surgically with a locking plate without allograft. For equal comparison, matches were made based on fracture characteristics, age (a range of 5 years) and American Society of Anesthesiologists classification.^1^ Regarding the fracture characteristics, matches were based on the following characteristics: Neer classification, fracture-involvement of anatomical neck, fracture-displacement, open fractures, and head split fractures. Patients were retrospectively selected from the electric health report system. Follow-up data were prospectively collected by clinical assessment, radiograph, and questionnaires. Inclusions and treatment were performed from 2017 until 2021 in four orthopedic trauma centers specialized in upper extremity injury (one level I trauma center and two level II trauma centers). All data were collected at one-year follow-up at the outpatient clinic or by house visits.

The study was approved by the medical ethical board of the Amsterdam University Medical Centre (020.541).

### Primary outcome measures

The primary outcome measures for this study are the clinical reported outcomes, patient-reported outcomes measures (PROMs), including Visual Analog Scale for pain, Disability of the Arm Shoulder and Hand (DASH) score, Constant Shoulder Score (CSS), and Oxford Shoulder Score (OSS).^5^^,^^15^^,^^29^ For reference values, Minimal Important Change (MIC) measurements in literature for these PROMs were used. The range of motion (ROM) was measured by forward flexion, external rotation, and abduction using a goniometer. All data were collected after one-year follow-up.

### Secondary outcome measures

Secondary outcomes consist of radiological union rate and development of AVN of the humeral head. Radiographic images were assessed at follow-up for fracture union with specific attention to the greater tuberosity and evaluation of signs of AVN. Furthermore, the head-shaft angle was measured on the anterior-posterior radiographs perioperative, postoperative at 6 weeks, 6 months, and 12 months. Complications with a minimum grade III according to the Clavien-Dindo classification were recorded in each group.^11^

### Surgical procedure and follow-up

**Figure 1:**
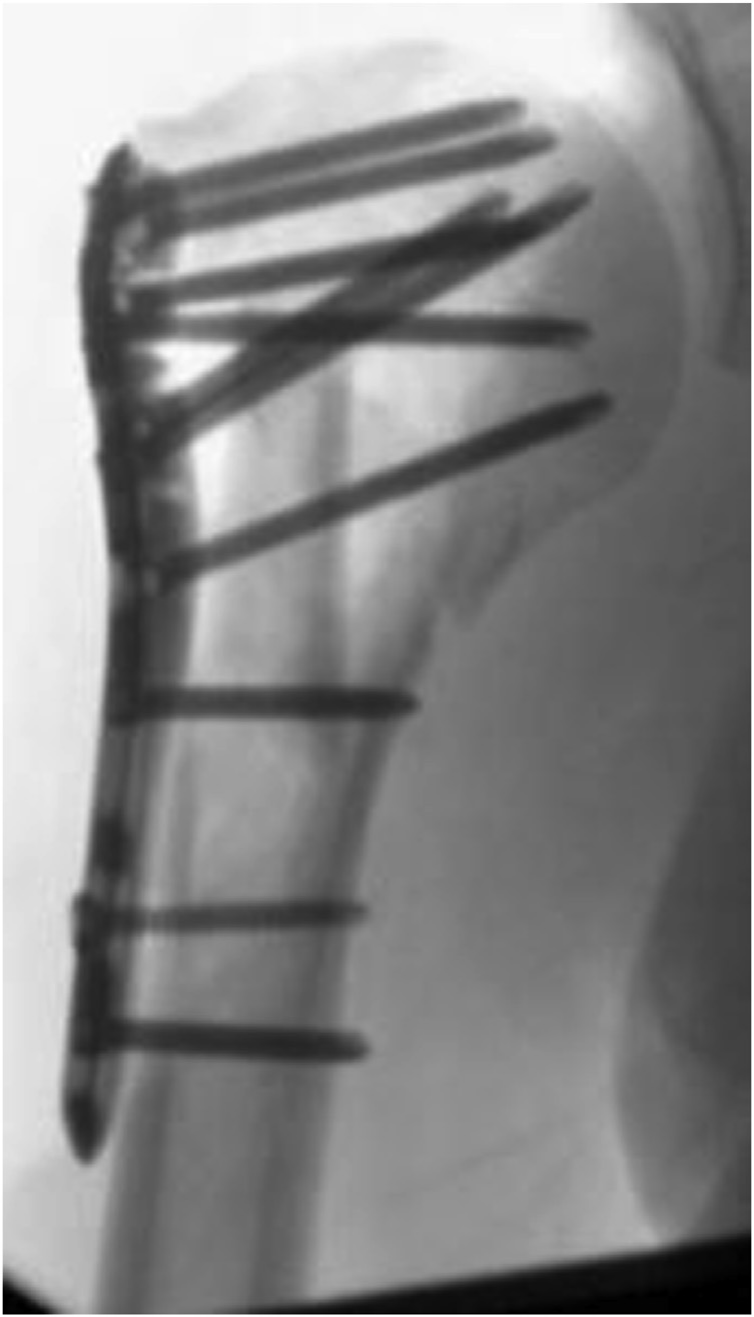
Intramedullary.

**Figure 2:**
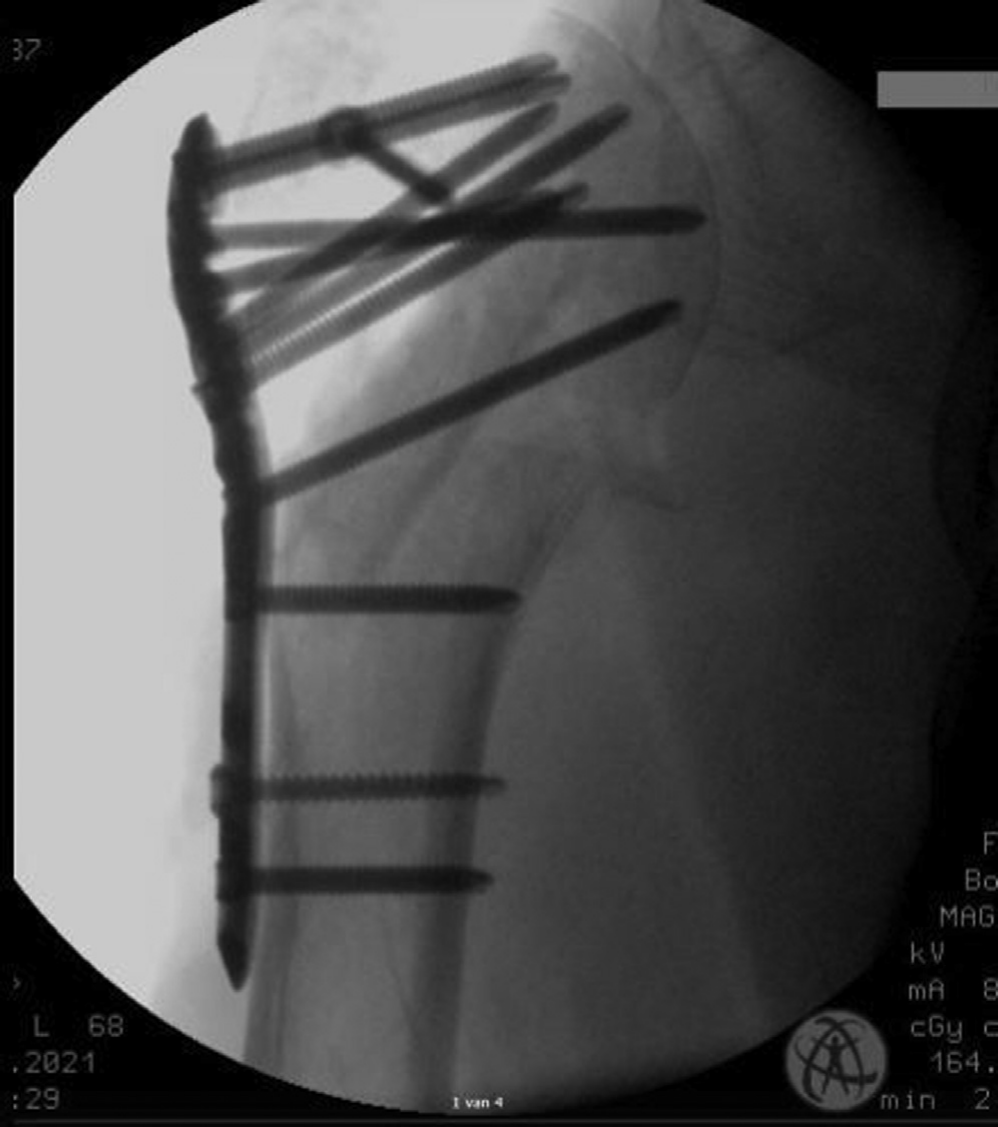
Transversal.

The surgical procedures were performed by three orthopedic trauma surgeons specialized in shoulder surgery, working according to the same operating principles in the same shoulder surgery network. For all patients the deltopectoral approach was used. Surgery was performed within one month after trauma, under general anesthesia combined with plexus blockade in beach-chair position and with the use of intra-operative fluoroscopy. For the locking plate osteosynthesis, the PHILOS System from DePuySynthes (Raynham, MA, USA) was used, or the Carbofix (Carbofix Traumasysteem; Oudshoorn, South Africa). Fibula allografts were collected from the Dutch bone bank and positioned as intramedullary pegs (Fig. 1) or transversal strut (Fig. 2) for the medial hinge. Allografts were fully denaturalized before implantation.

Postoperative physical therapy included the start of circumferential exercises in the first week, progressing to 90° abduction in the next five postoperative weeks guided by a specialized shoulder physical therapist. Standard follow-up examinations including measuring the ROM and pain scores were performed at two weeks, six weeks, three months, and one year following treatment. Six weeks postoperatively, standard shoulder radiographical images were performed to assess secondary displacement. At one-year follow-up, radiographic imaging was performed to objectify union and to ascertain whether AVN was present. In addition, CROs and PROMs were obtained at this visit.

### Statistical analysis

Descriptive statistics were used to present patient and fracture characteristics. Normal distribution of the data was determined by the Shapiro-Wilk test. For normally distributed data, the independent students' *t*-test and chi-squared tests for continuous and categorical variables were used, respectively. For nonnormally distributed data continuous independent groups, the Mann-Whitney U test was used. *P* values <.05 in two-sided tests were considered statistically significant. All analyses were performed using IBM SPSS Statistics 23 (IBM Corp., Armonk, NY, USA).

## Results

A total of 13 patients who underwent locking plate osteosynthesis with fibula allograft augmentation met the inclusion criteria and received an invitation to participate in the study. From one patient, no response was received. Twelve patients (nine women) with a mean age of 58 years (SD 6.0) at the time of the injury responded and were enrolled. The control group consisted of 12 patients (seven women) with a mean age of 61 years (SD 4.8) (Tables I and II).

**Table I:** Comparison of demographic data between the two groups of patients.

Variables	Fibula allograft group (n = 12)	Control group (n = 12)	*P* value
Sex, male	3	5	
Mean age, y	58 (SD 6.0)	61 (SD 4.8)	
Neer classification			
Neer 3	5	4	
Neer 4	7	8	
Follow-up, mo			
Clinical	44 (16-66)	71 (21-133)	
Radiological	13 (9-33)	34 (5-192)	
Complications	3 (25%)	7 (58%)	.214
Consolidation	11 (92%)	8 (67%)	.317

*SD*, standard deviation.

Fibula group: plate osteosynthesis combined with fibula allograft; Control group: plate osteosynthesis alone.

**Table II:** Open-source data regarding matched patient and fracture characteristics.

Fibula strut group	Age	ASA	Neer	Fracture type	Control group	Age	ASA	Neer	Fracture type
1001	65	1	4	Anatomic neck	1101	69	2	4	Anatomic neck
1002	54	2	4	Anatomic neck	1102	59	1	4	Anatomic neck
1003	56	1	4	Headsplit + anatomic neck	1103	61	1	4	Headsplit + anatomic neck
1004	55	1	3	Anatomic neck	1104	56	1	4	Anatomic neck
1005	52	1	3	Gustillo 2, anatomic neck	1105	62	1	4	Posterior luxation, anatomic neck
1006	63	2	4	Anatomic neck	1106	64	2	4	Anatomic neck
1007	70	2	3	Anatomic neck	1107	65	2	3	Anatomic neck
1008	53	2	4	Head split	1108	56	2	4	Head split
1009	53	2	3	Anatomic neck	1109	57	2	3	Anatomic neck
1010	52	2	3	Anatomic neck	1110	58	2	3	Anatomic neck
1011	63	2	4	Anatomic neck	1111	67	2	4	Anatomic neck
1012	60	3	4	Anatomic neck	1112	54	3	4	Anatomic neck

*ASA*, American Society of Anesthesiologists.

Patient and fracture characteristics with plate osteosynthesis + fibula allograft vs. plate osteosynthesis alone.

### Patient reported outcomes

Statistically and clinically relevant difference (exceeding the MIC for the score used, shown in Table III^21^^,^^35^^,^^36^) was found for the DASH score in favor of the fibula group. For the DASH, a mean difference of 8.7 was found (*P* = .048), the CSS had a mean difference of 3.3 (*P* = .040). The CSS did not exceed the MIC (Table III^22^). The OSS score did not reach statistical significance (*P* = .105). The VAS was not significantly different between both the groups (Table III^31^).

**Table III:** Patient reported outcome measures.

PROMs	Fibula group (n = 12) Mean ± SD	Control group (n = 12) Mean ± SD	Mean difference	*P* value	Reference MICs
VAS	3.1 ± 1.8	1.6 ± 1.9	1.5	.439	2.4
DASH	17.4 ± 8.6	26.1 ± 19.2	8.7	.048	8.1-13
CSS	16.5 ± 11.5	19.8 ± 16.5	3.3	.040	5.4, 11.6, 17.7
OSS	40.2 ± 6.6	38.3 ± 9.5	1.9	.105	5.1, 11.4

*PROMs*, patient reported outcome measures; *VAS*, Visual Analog Scale; *DASH*, disability of arm-shoulder-hand score; *CSS*, Constant-Murley score; *SD*, standard deviation; *MIC*, minimal important change.

Fibula group: PHILOS, combined with fibula allograft; Control group: PHILOS, alone.

### Range of motion

For the ROM, a significant difference was found for abduction and external rotation in favor of the fibula group (respectively 127° vs. 92°, *P* = .045 and 50° vs. 26°, *P* = .028) (Table IV).

**Table IV:** Range of motion.

ROM	Fibula group (n = 12)				Control group (n = 12)				*P* value
	Min	Max	Mean	Standard deviation	Min	Max	Mean	Standard deviation	
Flexion (°)	75	180	132	37	20	164	102	46	.114
Abduction (°)	70	180	127	38	24	180	92	49	.045
External rotation (°)	15	90	50	21	0	54	26	23	.028

*ROM*, range of motion.

Fibula group: PHILOS, or carbofix combined with fibula allograft; Control group: PHILOS, alone.

### Radiological outcome measures

Radiographic union was reached in eleven patients (92%) of the fibula group compared to eight (67%) in the control group (*P* = .317, Supplementary Table S1). The difference in preoperative and postoperative head-shaft angle in the fibula group was 2° ± 4°, whereas in the control group, the angle difference was −3° ± 14° (*P* = .237).

### Complications

Because of the limited numbers of the groups the complications were described in percentages rather than in significance.

There were three postoperative complications (25%) in the fibula group. Two patients developed AVN 1.5 and 2 years after surgery respectively, for which they were treated with an RfSA. Patient 1011 developed a screw cut out without AVN but never returned for removal of the implant.

In the control group, there were seven postoperative complications (58%) of which four patients developed AVN. Patient 1101 developed a superficial wound infection at first and AVN in a later stage. Patient 1103 developed AVN after valgus collapse after 14 months. Patient 1105 developed AVN after five months. Patient 1108 developed deep venous thrombosis. Patient 1109 developed a deep infection and AVN after five months. Patient 1111 developed screw penetration after six months. An appointment was made to remove the screws however the patient failed to return to the outpatient clinic. Patient 1112 developed permanent nerve damage of a side branch of the median nerve after which full flexion of the fingers was limited.

## Discussion

This multicenter, matched cohort study showed favorable outcomes of fibula allograft augmentation of locking plate osteosynthesis for patients with complex PHFs. After fibula allograft augmentation, patients showed significantly better DASH scores, also exceeding MIC values. In addition, abduction and external rotation were significantly better in the patients following fibula allograft augmentation. Less complications were recorded in the fibula group. Therefore, we state that the use of a fibula allograft in selected complex PHFs results in better clinical outcomes with lower complication rates.

According to the DASH score and CSS, a significant difference in favor of the fibula allograft group was found with differences of 8.7 and 3.3 points, respectively. When comparing these outcomes to literature reporting on PROMs, an MIC between 8.1-13.0 has been reported on the DASH was reported for the patients with PHFs.^14^ This strengthens our conclusion that not only a statistical significant, but more importantly a clinical relevant difference is present in DASH scores when augmenting complex PHFs with fibular allografts. It needs to be taken into account that the MIC is a measurement of outcomes within one patient and not between cohorts. However, due to the matched-control nature of this study, correction for important possible influencing factors on MIC has been performed.

Regarding the ROM, external rotation and abduction showed better results in the fibula group. This may be because fibula allograft augmentation provides better support of the injured shoulder joint. As described before, the fibula allograft placed as a medial strut across the fracture supports the articular head. This allows for a more stable construct and prevents malalignment.^3^^,^^16^^,^^25^^,^^9^^,^^38^ The current study also demonstrates these positive effects of strut placement.

When comparing our results to the available literature, Zhao et al reported head shaft angles following fibula allograft augmentation of 136° vs. 126° in the control group, with significant difference (*P* < .001), as well as Cui et al showing a mean head-shaft angle difference of 3° vs. 10° (*P* < .001).^12^^,^^38^ Davids et al were not able to show this difference, most likely due to heterogeneity between groups in fracture types.^13^ The results from both Zhao et al and Cui et al endorse our findings that the head-shaft angle is more stable with the use of an allograft augmentation and therefore reconstruction with fibula allograft for an anatomical neck fracture may be beneficial. This is especially true for young and active patients where head-preserving therapy is preferable over treatment with arthroplasty.

In this matched cohort study, the overall complication rate was more than double in the control group (25% vs. 58%). Compared to other studies reporting complication rates of greater than 35% after surgery for PHFs,^10^ the complication rate in the fibula group was much lower. Infection, screw penetration, and AVN occurred more often in the control group. Furthermore, we determined less varus collapse of the medial hinge in the fibula group compared to the control group. This implies that fibula strut augmentation indeed is a safe and beneficial addition with lower risk of complications for the surgical treatment of complex PHFs. Moreover, the lower rate of complications combined with improved mobility could result in lower health care costs as there is likely less need for outpatient clinic visits, reoperations, and home care.

AVN occurred in two patients in the fibula group compared to four patients in the control group. The primary goal of this treatment is to prevent active patients from losing their joint due to AVN, therefore this is clinically a very relevant finding. The time to develop AVN following locking plate osteosynthesis appears to be around six months, while we also found late AVN after one and a half years. This implies the need for prolonged follow-up for these type of high-risk fractures or accessible return to health care facilities when patients experience loss of function or increasing pain.

### Strength and weaknesses

To our knowledge, this is the first study to specify indication criteria for the use of fibula allograft and describe a matched case-control cohort based on these indications. Using this allograft specifically for relatively young and active patients with complex 3-part or 4-part fractures with anatomical neck participation sheds better light on the biomechanical adjunct of the allograft.

The number of 24 patients may have been insufficient to reach statistical significance and draw firm conclusions. Further (prospective) studies with a greater number of patients are needed. One might argue that matching should have been performed on sex. However, since osteoporosis occurs at a higher age and more often in women,^6^ in these relatively young patients sex was not a criterium. According to the complexity and potential implications of osteonecrosis we might have considered a longer follow-up period (eg, 2 years).

### Clinical implications

Taking the limitations of this study into account, we did show that with specific selection of complex fracture types and patient characteristics, better outcomes in PROMs, ROM, and fewer complications were seen when a fibula allograft was used. Selection criteria for fibula strut augmentation are essential. The current available literature describes heterogeneous fracture classifications in elderly patients. Two studies only analyze the use of the fibula allograft in complex PHFs, although in an elderly population, and present similar favorable outcomes of the use of a fibular strut as seen in our study.^12^^,^^38^ Another study describing a heterogeneous fracture classification (2-part and 3-part) in younger patients, was not able to find the same favorable outcomes.^13^ Recently, for elderly patients with complex PHFs, arthroplasties are recommended due to the high complication rates in primary plate osteosynthesis.^2^ Nonetheless, two studies did include elderly patients and reported better functional outcome, PROMs and diminished complication rates after reconstruction with a fibula allograft. A recent randomized controlled study showed that using a fibula allograft did not result in better outcomes or less complications.^34^ However, this study only included patients with medial comminuted fractures in elderly patients and also included fracture type Neer 2. Moreover, the study compared heterogeneous groups (fibular allograft vs. locking plates) whereas our study matched cases one on one.^34^ We specifically included patients with anatomical neck fractures which are more likely to collapse. This might exactly be the reason why we found better outcomes in the fibula group. Moreover, in our study a physical therapist was consulted postoperatively and was closely involved in our patients aftercare, whereas patients in the randomized controlled trial were given only encouraging instructions, this also might have had a beneficial effect on our outcomes.

### Future perspectives

In general, a prospective study with a larger sample size should be performed to confirm our conclusions on indications for fibula allograft augmentation of locking plate constructs in complex PHFs. Also, future studies should focus on the optimal position of the fibula allograft in the proximal humerus and optimization of the biomechanical properties of the strut.

## Conclusion

Active patients with complex 3-part and 4-part PHFs including an anatomical neck fracture, benefit from locking plate fixation in combination with fibula allograft augmentation. Abduction and external rotation were statistically significantly better when fibular allograft was used. For PROMs statistically significant differences and clinically relevant differences were found for the DASH in favor for the fibular allograft. In addition, statistically significant better outcomes on CSS score were reported, not exceeding the MIC, in favor of the fibular allograft. Larger randomized trials will improve the power of these comparisons and may demonstrate convincingly that fibula grafts improve the outcomes when surgically treating 3-part and 4-part PHFs.

## Disclaimers:

Funding: No funding was disclosed by the authors.

Conflicts of interest: LS Blaas has an unrestricted educational grant by Mathys Medical Ltd. The other authors, their immediate families, and any research foundation with which they are affiliated have not received any financial payments or other benefits from any commercial entity related to the subject of this article.
