# Supplementary material for: Fibula allograft in complex three-part and four-part proximal humeral fractures in active patients, a matched case-control study
Source: JSES Int. 2023 Nov 17;8(1):21–6. doi: 10.1016/j.jseint.2023.10.004 (PMC10837717; doi:10.1016/j.jseint.2023.10.004)
Supplement: Supplementary Table S1 [file mmc1.docx]

**Appendix 1.** Open source data on radiographic outcomes and complication

| **Pt nr** | **Union (yes/no)** | **HSA Per-op**  **(**°**)** | **MH 6 wk**  **(**°**)** | **MH 6 mth**  **(**°**)** | **MH 1yr**  **(**°**)** | **Complications** |
| --- | --- | --- | --- | --- | --- | --- |
| 1001 | Yes | 134 |  |  | na |  |
| 1101 | No | 125 | 125 | Complication |  | Wound infection (2wks) and AVN (6mnths) |
| 1002 | Yes | 144 |  |  | 140 |  |
| 1102 | Yes | 127 | 107 |  | 108 |  |
| 1003 | Yes | 114 | 117 |  | 120 |  |
| 1103 | No | 151 | 151 |  | 166 | AVN (14mnths): valgus collaps |
| 1004 | Yes | 110 | 115 |  | 115 |  |
| 1104 | Yes | 130 |  | 130 | 127 |  |
| 1005 | No | 135 | 136 |  | 140 | AVN (1.5 yr) |
| 1105 | No | 141 | 105 | 103 | Complication | AVN (5mnths) |
| 1006 | Yes | 136 |  |  | 134 |  |
| 1106 | Yes | 113 |  |  | 115 | Nerve damage (median) |
| 1007 | Yes | 116 | 112 |  | 112 |  |
| 1107 | Yes | 122 | 124 |  | 125 |  |
| 1008 | Yes | 125 | 112 |  | 130 |  |
| 1108 | Yes | 117 | 126 |  | 122 | DVT |
| 1009 | Yes | 114 | 126 |  | 117 |  |
| 1109 | No | 136 | 138 | 128 | Complication | Deep infection (3wk) + AVN (5mnths) |
| 1010 | No | 133 | 130 |  | 130 | Impingement (6mnths), + AVN (2yr) |
| 1110 | Yes | 150 |  |  | 151 |  |
| 1011 | Yes | 160 | 163 | 164 | 166 |  |
| 1111 | No | 135 | 141 | 151 | Complication | Screw penetration (6mnths) |
| 1012 | Yes | 141 |  | 150 | Na | Screw penetration (13mnths) |
| 1112 | Yes | 138 |  | 132 | 123 |  |

Na: not available. HSA: Head-Shaft Angle
